# Nitrogen deficiency in maize: Annotated image classification dataset

**DOI:** 10.1016/j.dib.2023.109625

**Published:** 2023-09-27

**Authors:** Miroslav Salaić, Filip Novoselnik, Ivana Podnar Žarko, Vlatko Galić

**Affiliations:** a Agricultural Institute Osijek, HR31000 Osijek, Croatia; b Protostar Labs d.o.o.^1^, HR31551 Belišće, Croatia; c University of Zagreb^2^, Faculty of Electrical Engineering and Computing, HR10000 Zagreb, Croatia

**Keywords:** Computer vision, Precision farming, Agricultural robotics, Maize

## Abstract

Nitrogen (N) is one of the key inputs in maize production applied in the form of fertilizers. Nitrogen deficiency during the vegetation period leads to lower yields since N is utilized in proteins and enzymes that enable important biochemical processes such as photosynthesis. Nitrogen deficiency leads to specific symptoms that eventually become visible to the naked eye during vegetation. Our hypothesis was that N deficiency can be detected from maize RGB images in parametric process such as a deep neural network. The aim of the reported dataset is to optimize the usage of N in the farmer's fields and accordingly, reduce its environmental footprint. This dataset contains 1200 images of maize canopy from field trials, annotated by an expert from an agricultural institution. The field trials included three levels of N fertilization: N0 without N fertilization, N75 with 75 kg of added N fertilizer, and NFull with 136 kg of added N fertilizer. For each fertilizer level, 400 plots were created with 238 different maize genotypes, resulting in a total of 1200 plots. Images were taken with a tripod mounted DSLR camera, aperture priority set to f/8 and sensor sensitivity set to ISO400. Images were taken at a 45° angle to each plot. This dataset can be useful to both researchers, data scientists and agronomists, especially in the context of emerging technologies in precision agriculture, such as robotics, 5G networks and unmanned aerial vehicle (UAV). The dataset is one of the first publicly accessible datasets of maize canopy images under different N fertilization levels and represents a valuable public resource for development of machine learning models for in-season detection of N deficiency in maize.

Specifications Table

**Table:** 

Subject	Applied Machine Learning, Computer Science, Agricultural Science
Specific subject area	Computer vision techniques for the classification of nitrogen (N) deficiency in maize
Type of data	Image
How the data were acquired	Data collection was carried out with Canon 80D camera with an18–50 mm lens at 18 mm. Aperture priority mode was used with aperture set to f/8. The focus was set manually to approx. 1 m into the stands. The sensor sensitivity was set to ISO400. The images were taken using a tripod with 45° angle view on the plot.
Data format	JPG
Description of data collection	Images were collected in the field in July 2023, in a 5-day window around flowering time of different genotypes. There were three N fertilization levels: N0, N75 and NFull, with no added N, 75 kg of added N and full fertilization with 136 kg added N, respectively. 238 genotypes were sown in augmented design, where some genotypes are replicated, and the others are not. Images at different field rows were taken randomly between 7:30 and 11:00 a.m.
Data source location	• Institution: Agricultural Institute Osijek (AIO) • City/Town/Region: Osijek • Country: Croatia
Data accessibility	Repository name: Mendeley Data
	Data identification number: 10.17632/g7xnn2bm4g.1
	Direct URL to data: https://data.mendeley.com/datasets/g7xnn2bm4g/1 Instructions for accessing these data: Data are freely and anonymously downloadable from the link. Images are compressed into a single .zip file. Additionally, iPython notebook ‘TensorFlow_preprocessing.ipynb’ and ‘requirements.txt’ cover data preprocessing and required libraries to run the scripts.

## 1 Value of the Data

• The dataset of maize canopy images under different N fertilization levels can be used to train machine learning models to detect N deficiency in maize during flowering.
• The image dataset can be used by research scientists in field of agronomy for technological advancement of precision agriculture for maize production. This could also positively affect the N pollution, as N application would take place when needed, where needed.
• The image dataset can also be used for maize plant recognition in the field of computer vision and implementation of novel agrotechnical solutions, e.g., self-driving field robots, as it represents a real-life example of field crop trial.
• The image dataset might be useful for the extraction of RGB vegetation indices.
• Data is balanced and orthogonal, meaning that the plot represented in image label “N0 (199).JPG” with label N0 and number 199 represents the same plot from the design as for example “N75 (199).JPG”.
• Additionally, the data can be used to train models for weed recognition in maize crop.

## 2 Objective

Nitrogen fertilization is one of the most expensive inputs in maize production. It relies heavily on the use of energy in the Haber-Bosch process [1], most of which is obtained from the combustion of natural gas. In addition, nitrate, and nitrite leakage is known to cause a variety of medical conditions [2] and damage to natural ecosystems. With advancements in plant phenotyping [3], [4], [5] and its implementation in the fields of communications, electronics, and robotics [6], computer vision plays a key role in further progress of precision agriculture for maize production. Our dataset represents an attempt to detect in-season N deficiency from images of plant canopies. For example, there are some well-known indices that can be easily extracted from RGB reads [7], [8], [9] that are useful in areas of stress phenotyping. Our intention was to create a dataset that can be used for training computer vision models in the fields of plant science, agronomy, computer vision, and robotics.

Accordingly, the hypothesis of this research was that the maize agronomic indices over different N fertilization scenarios could be extracted from RGB images in parametric processes of machine learning. The main motivation for building such models is to optimize fertilization in both temporal (apply when needed) and spatial (apply where needed) terms using self-driving field robots or semi-autonomous machines. The aim of such endeavor is to optimize the usage of N in the farmer's fields and accordingly, reduce its environmental footprint.

## 3 Data Description

**Fig. 1:**
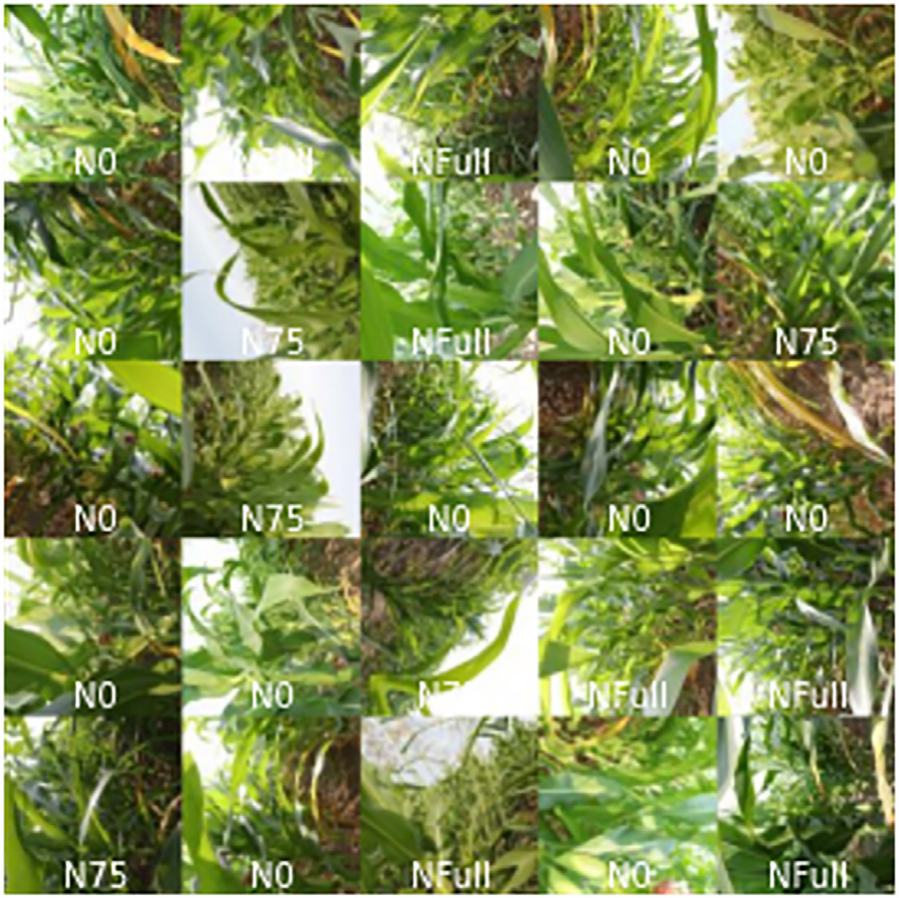
Mosaicked random sample of images from the full augmented dataset.

This dataset provides maize canopy images from field trials annotated by a PhD student from an agricultural institution (Fig. 1). The field trial covered three N fertilization levels: N0, N75, and NFull. The data is available in JPG format and was acquired using a Canon 80D DSLR camera with an 18–50 mm lens at 18 mm (∼29 mm in full frame equivalent). The maize N deficiency dataset can be used to train machine learning models to detect N deficiency in maize during flowering. Raw annotated data is available as a single folder at: https://doi.org/10.17632/g7xnn2bm4g.1, along with a preprocessing iPython notebook ‘TensorFlow_preprocessing.ipynb’. The notebook contains the functions with Docstring documentation used to preprocess the images. Furthermore, the notebook implements TensorFlow preprocessing [10] with a single level of augmentation, where the number of images in the training set is doubled using augmentation methods such as horizontal or vertical flip (Fig. 1). In addition, the notebook contains code needed to split the dataset to test and training subsets, while retaining balance of the label classes.

**Fig. 2:**
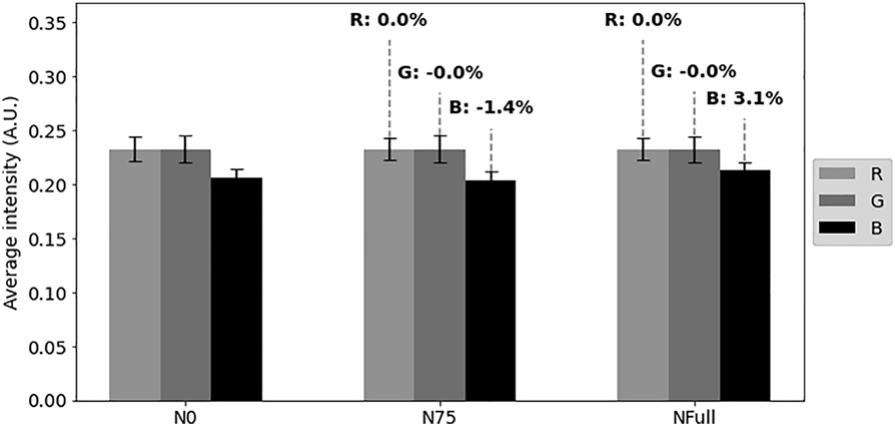
Average intensities of red (R), green (G) and blue (B) channels. Guided labels in N75 and NFull classes show percent average change per channel compared to N0.

Except visible features, N deficiency is expected to cause a spectral response (Fig. 2), detectable through vegetation indices calculated from changes in hue [11].

## 4 Experimental Design, Material and Methods

### 4.1 Experimental design

Totally 238 genotypes were sown in three experiments in augmented design, where some genotypes are replicated while others are not. Single N treatment was considered in a single experiment: N0, N75, and NFull, with no added N fertilizer, 75 kg/ha of added N, and full N fertilization with 136 kg/ha added N, respectively. All other agrotechnical measures were carried out in alignment with expert agronomist advice for high-yield maize. There were 400 plots in each experiment, resulting in 1200 plots in total.

### 4.2 Imaging

Images were taken with a Canon 80D and an 18–50 lens with manual focus set to ∼1 m within stands, aperture priority set to f/8, and sensor sensitivity to ISO400. Image size was set to 3.9 megapixel and image quality to high. Images were taken using a tripod at an 45° angle to each plot. Data collection was conducted at July 2023 in a 5-day window around flowering time of different maize genotypes. Images within field-columns were taken randomly and ordered afterwards. In addition, some of the field columns were imaged in an east-south orientation, while the others were imaged in a south-east orientation.

### 4.3 Data processing

The dataset created was balanced and symetric, i.e., one image of each genotype was taken in each trial. In addition, the image names (for example “N0 (1)”) harbor annotation with “N0” representing experiment N0 and number in the format “(1)” representing plot no. 1. The attached notebook named ‘TensorFlow_preprocessing.ipynb’ loads the annotations from the described data format. Further, the notebook contains code that offers functionalities of data augmentation using *Keras* RandomFlip and RandomRotation methods. Also, additional step of data segmentation is implemented, where K-means algorithm is applied to pixel values, after which the image is converted back to TensorFlow tensors. Out of the box, augmentation and segmentation procedures offer dataset expansion by twofold to four-fold.

## Limitations

First limitation of this dataset is the presence of weeds at some experimental plots. However, as a real-world example, this is an expectable occurrence. Further, models trained using this dataset might be biased towards type of soil used in experiments (eutric cambisol). Also, dataset represents a small number of scenarios (three) which if extended might be worthwhile for increasing the sensitivity of the models.

## Ethics Statements

NA.

## CRediT authorship contribution statement

**Miroslav Salaić:** Formal analysis, Investigation, Writing – original draft. **Filip Novoselnik:** Formal analysis, Methodology, Software. **Ivana Podnar Žarko:** Conceptualization, Funding acquisition, Project administration. **Vlatko Galić:** Conceptualization, Data curation, Investigation, Methodology, Software, Writing – original draft, Writing – review & editing.

## Data Availability

Nitrogen deficiency in maize: annotated image classification dataset (Original data) (Mendeley Data)
